# SOX3 expression in the glial system of the developing and adult mouse cerebellum

**DOI:** 10.1186/s40064-015-1194-1

**Published:** 2015-08-07

**Authors:** Pike-See Cheah, Paul Q Thomas

**Affiliations:** Discipline of Biochemistry, School of Molecular and Biomedical Sciences, University of Adelaide, Adelaide, Australia; Department of Human Anatomy, Faculty of Medicine, Health Sciences, University Putra Malaysia, 43400 Serdang, Selangor Malaysia; Neurobiology and Genetics Group, Genetics and Regenerative Medicine Research Center, Faculty of Medicine and Health Sciences, University Putra Malaysia, 43400 Serdang, Malaysia

**Keywords:** Transcription factor, Cerebellum, Glial cells

## Abstract

**Background:**

The cerebellum plays a vital role in equilibrium, motor control, and motor learning. The discrete neural and glial fates of cerebellar cells are determined by the molecular specifications (e.g. transcription factors) of neuroprogenitor cells that are influenced by local microenvironment signals. In this study, we evaluated the expression and function of *Sox3,* a single-exon gene located on the X chromosome, in the developing cerebellum.

**Result:**

In the embryonic and early postnatal cerebellum, SOX3-positive-cells were detected in the ventricular zone, indicating that SOX3 expression is present in a subset of the cerebellar precursor cell population. In the young adult cerebellum, this expression was diminished in cerebellar cells, suggesting its limited role in cerebellar progenitors. SOX3-positive-cells were also found in the cerebellar mantle zone. Further immunohistochemistry analyses revealed that SOX3 was not expressed in Purkinje neurons. Using glial markers in the early postnatal cerebellum, we found that virtually all of the SOX3-positive-cells were glial cells, although not all glial cells were SOX3-positive-cells. We also determined the impact of transgenic expression using a loss-of-function (Sox3 null) model. We did not observe any developmental defects in the cerebellum of the *Sox3* null mice.

**Conclusions:**

Our results indicate that the SOX3 protein is not expressed in cerebellar neurons and is instead expressed exclusively in the cerebellar glial system in a subset of mature glial cells. Although the expression of *Sox3* cerebellar glial development is lineage-restricted, it appears that the absence of *Sox3* in the ventricular germinal epithelium and migrating glia does not affect cerebellar development, suggesting functional redundancy with other SoxB1 subgroup genes.

**Electronic supplementary material:**

The online version of this article (doi:10.1186/s40064-015-1194-1) contains supplementary material, which is available to authorized users.

## Findings

### Background

*Sox3* is an X-linked member of the SOX gene family of transcription factors, which are defined by the presence of a *Sry*-related high mobility group that mediates sequence-specific binding to DNA (Collignon et al. 1996; Denny et al. 1992; Stevanovic et al. 1993). *Sox3* is expressed in progenitor cells throughout the entire neuraxis and is downregulated during neural differentiation (Brunelli et al. 2003; Collignon et al. 1996; Rex et al. 1997; Uwanogho et al. 1995; Wood and Episkopou 1999). Previous work has revealed that SOX3 regulation is region-specific in the developing nervous system, consistent with findings suggesting that it plays different roles in the dorsal telencephalon and hypothalamus (Rogers et al. 2013; Szarek et al. 2010). In humans, *SOX3* mutations and duplications are associated with X-linked hypopituitarism, a congenital male-specific syndrome that is characterized by pituitary hormone deficiency, infundibular hypoplasia, and incompletely penetrant mental retardation (Laumonnier et al. 2002; Solomon et al. 2002; Woods et al. 2005). Evidence across a variety of vertebrate species indicates that *Sox3* functions as a context-dependent regulator of cell differentiation. In mice, *Sox3* gain-of–function in the uncommitted XX gonad results in male sex reversal (Sutton et al. 2011), while loss-of-function in the postnatal testes germ cells blocks early spermatogenesis (Laronda and Jameson 2011). Together, these studies suggest an important role for *Sox3* in CNS neural development; however, the expression and function of *Sox3* in the cerebellum has not previously been reported.

The cerebellum, also known as “little brain,” represents only 10% of total brain volume but plays an important role in fine-tuning motor movement and balance. The cerebellum is derived from dorsal rhombomere 1 under the influence of the isthmic cells of the midbrain-hindbrain boundary (MHB) (Joyner 1996; Wingate and Hatten 1999; Wang and Zoghbi 2001). Previous studies have demonstrated that *Sox1* and *Sox2* are expressed in the Bergmann glial cells of the adult mouse (Sottile et al. 2006; Alcock and Sottile 2009) and human cerebellum (Alcock et al. 2009), although *Sox1* is not necessary for Bergmann glial cell generation in mice. To date, there are limited studies on *SoxB1* (and *Sox3*) expression in the embryonic and adult cerebellum.

## Methods

### Mice and tissue preparation

The timing of embryos was determined by using the day the vaginal plug was detected (timestamp: noon) as 0.5 days post-coitum (dpc) (n = 3 female mice for each time point). The timing of postnatal mice was defined by the day of birth, which was designated as postnatal-day 0 (P0). The *Sox3* null mouse strain has been described previously (Rizzoti et al. 2004). The Animal Ethics Committee of the University of Adelaide approved all animal experiments.

### Immunohistochemistry and microscopic analysis

Pregnant mice were sacrificed by CO_2_ inhalation followed by cervical dislocation prior to the removal of embryos. The embryonic and postnatal mouse brains were fixed with 4% paraformaldehyde in phosphate-buffered saline (PBS) overnight at 4°C, cryoprotected with 30% sucrose in PBS, and embedded in optimal cutting temperature (OCT) tissue embedding medium (Tissue-tek, Miles Laboratories). Tissue was cut into 12 and 18 µm sagittal sections for embryonic and adult samples, respectively, using Leica CM 1900 cryostat (Leica Microsystems, Inc., Bannockburn, IL).

The sections were blocked in 10% non-immune horse serum in PBST (0.1X PBS+0.3% Triton X-100) for 1 hour (h) at room temperature (RT) and subsequently incubated with primary antibodies overnight at RT. Following 3 washes in 0.1X PBS, the sections were incubated with secondary antibodies for 1 h at RT. The sections were then washed 3 times with 0.1X PBS and mounted in Prolong^®^ Gold antifade reagent with DAPI (Molecular Probes). The following primary antibodies were used: goat anti-SOX3 (1:100, R,D Systems), rabbit anti-Calbindin (1:1,000, Millipore), goat anti-GFP (1:400, Rockland), rabbit anti-SOX2 (1:1,000, Millipore), rabbit anti-SOX9 (1:1,000, gift from Professor Lovell-Badge), rabbit anti-GFAP (1:200, Sigma), rabbit anti-Ki67 (1:1,000, Novacastra), and rabbit anti-GLAST (1:1000, Abcam). The following secondary antibodies were used: donkey anti-goat IgG, Cy3 conjugated (1:400, Jackson ImmunoResearch Laboratories), and donkey anti-rabbit IgG, Cy5 conjugated (1:400, Jackson ImmunoResearch Laboratories). Immunofluorescence was observed using Zeiss Axioplan 2 fluorescence microscope (Carl Zeiss, Oberkochen, Germany). The micrographs were processed using AxioVision 4.7software (Carl Zeiss).

## Results

### SOX3 is expressed in the cerebellar ventricular zone and mantle zone of the developing mouse cerebellum

**Fig. 1:**
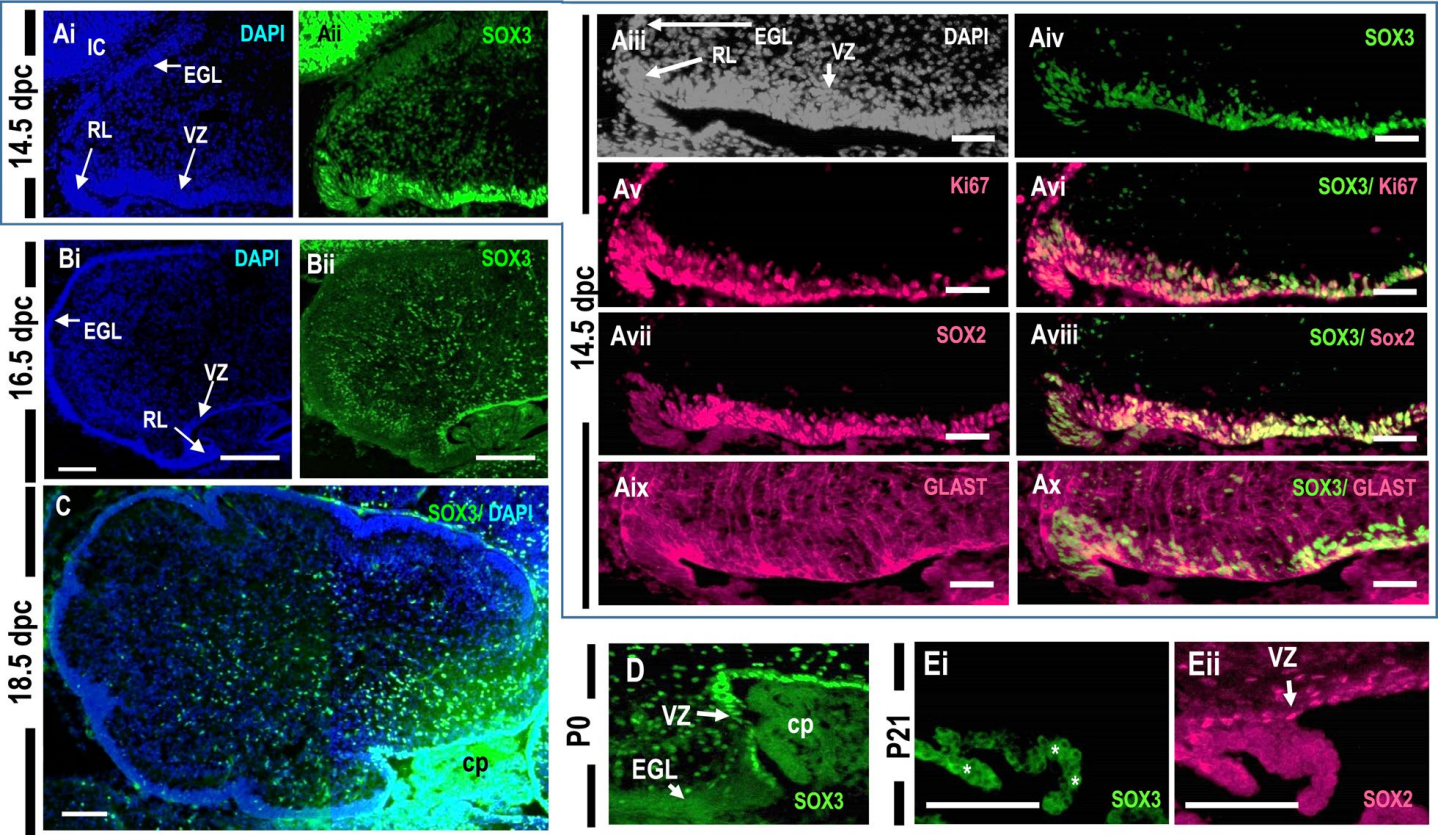
The SOX3 protein is expressed in the ventricular zones of the developing cerebellum but is absent in the adult ventricular zone. Sagittal sections of the cerebellum at 14.5 dpc (**A**), 16.5 dpc (**B**), 18.5 dpc (**C**), postnatal-day 0 (P0) (**D**), and P21 (**E**). **A**–**D** SOX3 signals are observed in the ventricular zone (VZ) and in the cerebellar mantle zone. **Aiii**–**Ax** In 14.5 dpc cerebella, SOX3 (*green*) expression overlaps with the Ki67+ proliferating cells, SOX2+ neural progenitors (*red*), and GLAST+ radial glial cells (*red*) [*yellow staining* in (**Avi**) (**Aiii**), and (**Ax**) respectively]. **E** Neural stem cells in the thin ventricular zone lining the fourth ventricle (4th V) of the adult cerebellum expressed SOX2 expression (*red*) but not Sox3 (*green*). *Autofluorescence of red blood cells within choroid plexus (cp). *Scale bar* for **Ai**, **Aii**, **C**–**E** = 100 um; **Aiii–Ax** = 500 µm.

At 14.5 dpc, SOX3-expressing cells were present primarily in the VZ; some SOX3-positive cells were also present in the mantle zone of the cerebellum (Fig. 1A). At 16.5, 18.5 dpc, and P0, SOX3-positive cells were dispersed throughout the cerebellar mantle zone and also present in the VZ (Fig. 1B–D). In the P21 cerebellum, the cells that remained in contact with the ventricular region expressed SOX2 protein (adult neural stem cell marker) but did not express SOX3 (Fig. 1Eii). These data indicate that SOX3 is expressed in mitotic progenitors of VZ in the developing cerebellum until the early postnatal stage, suggesting its potential role in early developmental stages of the cerebellum. The data for the young adult cerebellum also demonstrated that SOX3 plays a limited role in adult neural stem cells.

To more precisely characterize the SOX3 expression in the VZ, we carried out co-immunostaining of SOX3 with several molecular markers at 14.5 dpc cerebellum. Some SOX3-positive cells in the VZ (Fig. 1Aiii, iv) co-expressed Ki67 (a marker for proliferating cells) (Fig. 1Av, vi; Additional file 1: Figure S1), SOX2 (a marker of neuronal progenitors**)** (Fig. 1Avii, viii; Additional file 1: Figure S1), and GLAST/glutamate aspartate transporter (which marks radial glial progenitor cells) (Fig. 1Aix, x; Additional file 1: Figure S1), indicating that SOX3 expression at this stage marks a subset of the cerebellar precursor cell population.

### SOX3 is not expressed in cerebellar Purkinje neurons and it expression is glial-specific

**Fig. 2:**
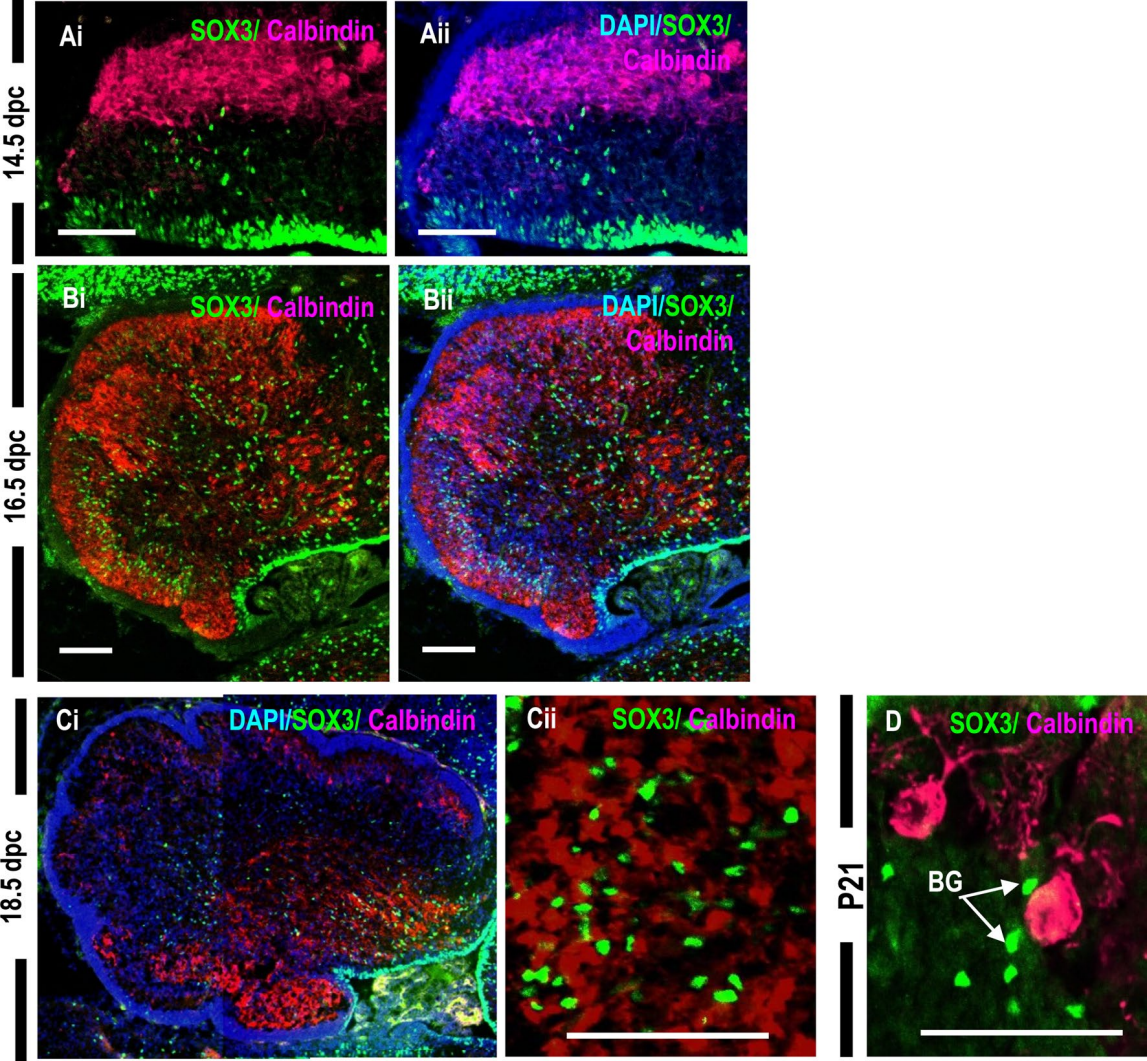
SOX3 is not expressed in the cells committed to cerebellar Purkinje neuron lineage. Sagittal sections of the 14.5 dpc (**A**), 16.5 dpc (**B**), 18.5 dpc (**C**), and P21 (**D**) cerebella. **Cii** Higher magnification of 18.5 dpc wildtype cerebellum showing that SOX3+ cells (*green*) are interpersed with Calbindin+ Purkinje cell (*red*). (**D**) Higher magnification reveals the close anatomical association between the SOX3+ cells (*arrows*) and PCs in P21 cerebellum. *BG* bergmann glial cells; *Scale bar* 100 µm.

To further investigate the lineage (glial versus neuronal) of the SOX3 positive cells in the mantle zone and parenchyma of the developing and early postnatal cerebellum, we utilized the Purkinje neuronal marker antibody Calbindin D-28k and also used a battery of radial glia/astrocyte marker antibodies, including anti-GLAST, anti-GFAP (glial fibrillary acidic protein; expressed in astrocytes and Bergmann glial cells, anti-SOX2 (Bergmann glial cell marker), and anti-SOX9 (glial marker). In between 14.5 dpc until 18.5 dpc, Calbindin-expressing PCs were distributed in a broad cellular zone within the cerebellar mantle zone, showing a superficial-deep gradient with many PCs concentrated in the cortical zone beneath the EGL (Fig. 2A–C). At 14.5 dpc, SOX3-expressing cells were present primarily in the VZ with a few SOX3-positive cells expressed in the mantle zone (Fig. 2A). At 16.5 dpc, cells were well dispersed throughout the cerebellar mantle zone (Fig. 2Bi, ii).

The SOX3-positive cell population was distinct from the postmitotic PC population (Fig. 2A, B). SOX3-expressing cells intermingled with the migrating PCs but never exceeded the front line of migrating PCs and were instead situated just beneath (Fig. 2A–C). At higher magnification of 18.5 dpc, SOX3-positive cells were intermingled but clearly distinct from the neurons that formed the Purkinje cell plates, indicating that the SOX3-positive cells are not post-mitotic Purkinje neurons (Fig. 2Cii). In P21 cerebellum, the somata of the smaller SOX3-expressing cells were clearly n in juxtaposition with larger PCs (arrows in Fig. 2D), suggesting that the SOX3-positive cells are indeed Bergmann glial cells.

**Fig. 3:**
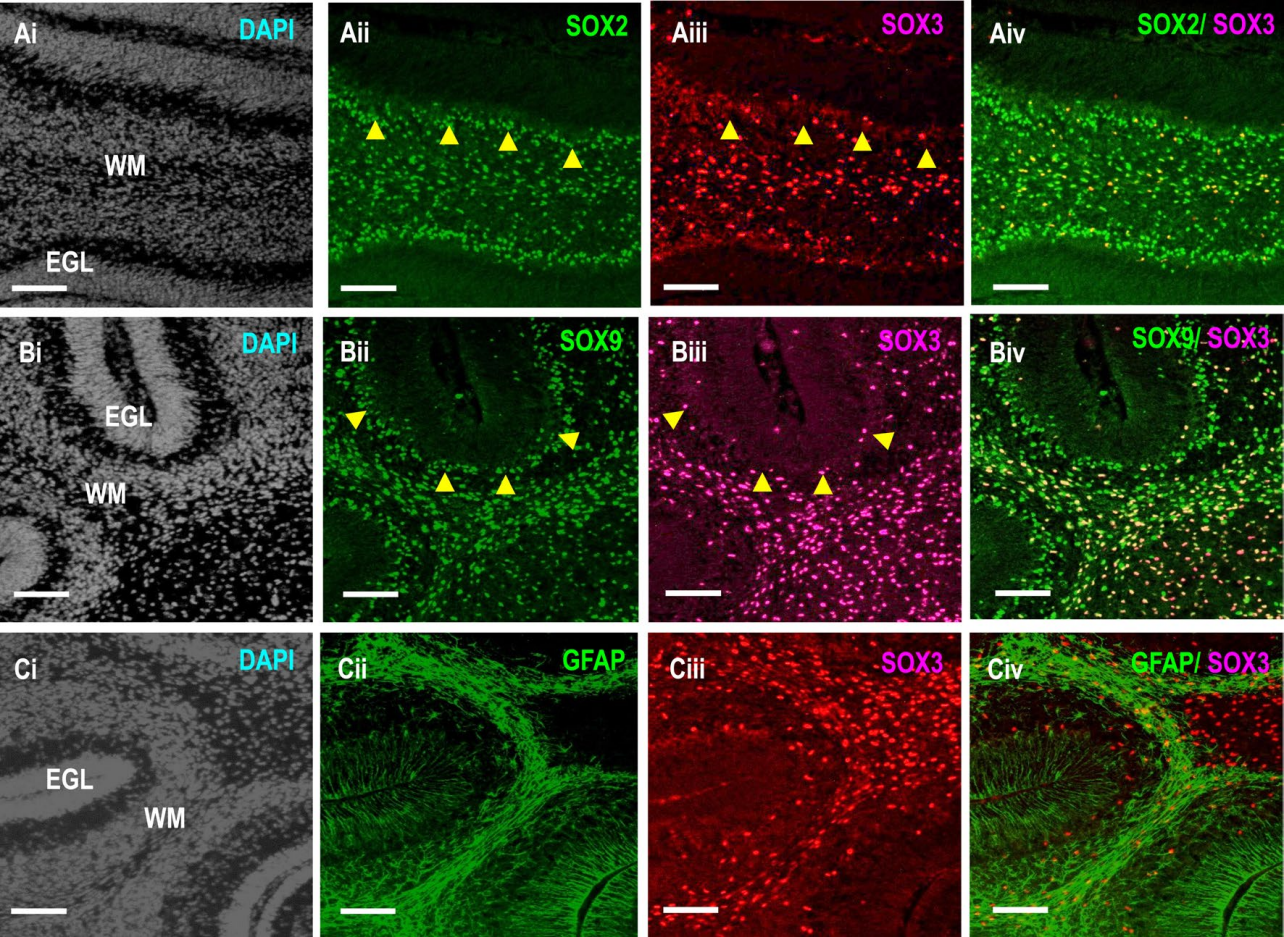
Sox3 expression is cerebellar glial-specific. **A**–**C** Sagittal sections of P7 cerebellum are co-stained with various glial markers including SOX2, SOX9 and GFAP (*green*), and SOX3 (*red*) and counterstained with DAPI (*blue*). Virtually, all SOX3+ cells are glial cells, although not all SOX2+, SOX9+, and GFAP+ glial cells coexpressed with SOX3 in the P7 cerebellum. *Scale bar* 500 µm.

SOX3-positive cells were present throughout the cerebellum during this stage. Since earlier data demonstrated that SOX3-positive cells are not committed to Purkinje neurons, we then confirmed their fate to become glial cells. Based on immunohistochemistry for SOX2, SOX9, and GFAP, SOX3 is expressed in a subset of astrocytes and Bergmann glial cells (yellow arrowheads; compacted Bergmann glial cells forming an epithelium-like lining in the PCL) (Fig. 3). We also observed that virtually all SOX3^+^ cells were glial cells, although not all SOX2^+^, SOX9^+^, and GFAP^+^ glial cells were SOX3^+^ in the early postnatal cerebellum (Fig. 2). In summary, the SOX3 protein is expressed exclusively in the cerebellar glial system in a subset of mature glial cells.

### SOX3 loss-of-function does not overtly affect cerebellar cell differentiation

**Fig. 4:**
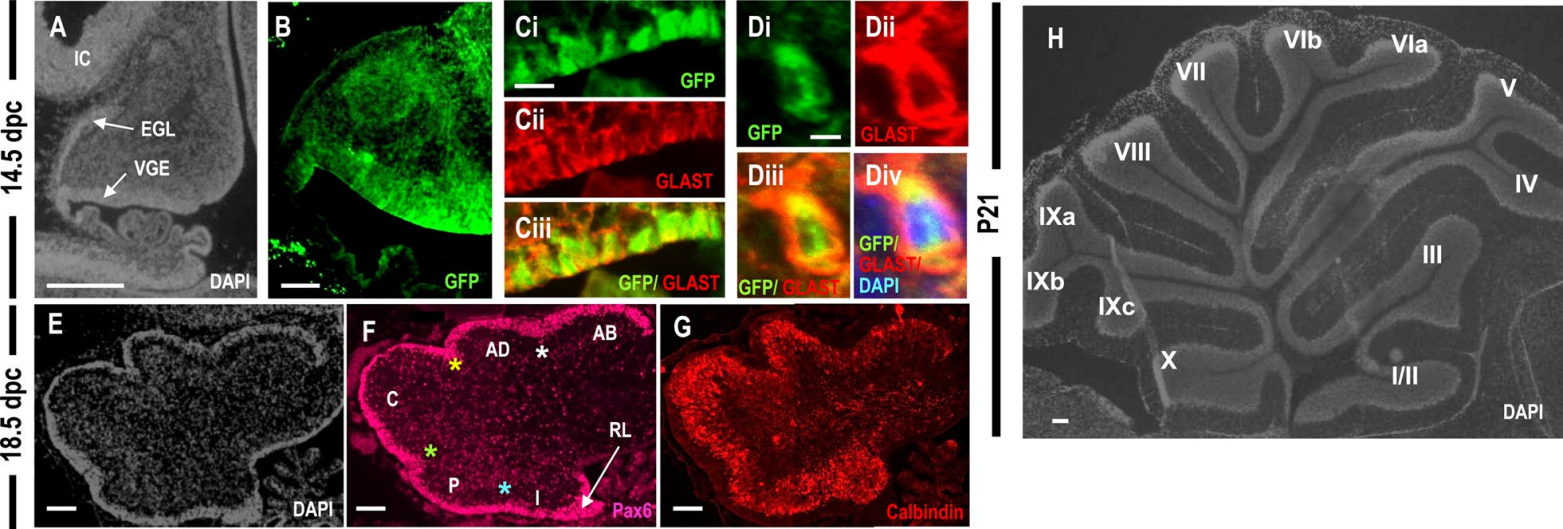
Normal cerebellar lamination and development in *Sox3* knockout mice. **A**–**D** Sagittal sections of 14.5 dpc of the Sox3 null cerebellum. **B** Expression of GFP+ cells (*green*) in the ventricular germinal epithelium (VGE). GFP+ cells are absence in the external granular layer (EGL). **C** Higher magnification of the ventricular germinal epithelial cells shows coexpression of GFP and GLAST. **D** Higher magnification view of a GFP+ cell that coexpressed GLAST. Sagittal section of the Sox3 null cerebella at 18.5 dpc. **E** Sagittal section of 18.5 dpc of the Sox3 null cerebellum. **F** Pax6 + GCPs (*red*) populating the EGL indented to form the four principal fissures (*asterisks*: *white* = preculminate fissure; *yellow* = primary fissure; *green* = secondary fissure; *blue* = posterolateral fissure) of the 18.5 dpc cerebellum. **G** Calbindin+ Purkinje cell plates foliate simultaneously at the base of the principal fissures. **H** Sox3 knockout mice have a normal size and well patterned cerebellum. *AB* anterobasal lobe, *AD* anterodorsal lobe, *C* central lobe, *P* posterior lobe, *I* inferior lobe. *Scale bar* 100 µm (**A**–**C**; **E**–**H**); 50 µm (**D**).

To determine whether *Sox3* is required for cerebellar glial cell formation, we analyzed *Sox3* null embryos containing a GFP knock-in reporter allele (Rizzoti et al. 2004). At 14.5 dpc, *Sox3* null embryos showed normal morphology of the cerebellar primordium (Fig. 4A). Consistent with the expression of SOX3 in the wild type cerebellum, GFP was detected in the ventricular germinal epithelium (Fig. 4B) and overlapped with GLAST (higher magnification in Fig. 4C, D) in Bergmann glial cells and migrating glia. At 18.5 dpc, DAPI, PAX6, and Calbindin immunostaining indicated that foliation was normal in the *Sox3* null cerebellum (Fig. 4E–G) suggesting that Purkinje cell plate (PCP) formation occurred normally in the absence of functional Sox3 protein in the cerebellar glial cells (Fig. 4G). After birth, the GFP signal was not detectable in the *Sox3*-null cerebellum (Additional file 2: Figure S2). Analysis of P21 *Sox3* null mice revealed normal development, foliation, and lobulation of the cerebellar cortex (Fig. 4H). In summary, despite the lineage-restricted expression of SOX3 cerebellar glial development, the absence of SOX3 in the ventricular germinal epithelium and migrating glia does not impair the development, foliation, and lobulation of the cerebellum.

## Discussion

*Sox3* and the other *SoxB1* family members, *Sox1* and *Sox2*, are expressed in neural progenitor cells in the developing CNS (Bylund et al. 2003; Collignon et al. 1996; Rogers et al. 2013). Recent reports have shown that SOX3 is also expressed in the developing telencephalon, hypothalamus, and differentiated hypothalamic neurons throughout adulthood (Rogers et al. 2013; Szarek et al. 2010; Wang et al. 2006) and in the subcommissural organ (Lee et al. 2012), indicating that its expression is region-specific and that it plays distinct roles in different parts of the brain. SOX3-positive cells have also been detected in the subventricular zone of the cerebral cortex and in the hippocampal subgranular zone of the adult mouse brain (Wang et al. 2006).

In this study, we found that SOX3 was expressed in the ventricular zone, as well as in some cerebellar cells in the mantle zone, during the embryonic and early postnatal stages of cerebellar development. The cerebellar ventricular zone is an important germinal center that gives rise to many types of cerebellar cells, including inhibitory Purkinje cells, cerebellar glial cells, deep cerebellar nuclei, Golgi interneurons, and the precursors for inhibitory cerebellar cortical interneurons (basket and stellate cells). Our data showed that SOX3 is expressed in mitotic progenitors of the VZ in the developing cerebellum, indicating its potential role in giving rise to other cerebellar cells derived from the ventricular zone. Our data also revealed that SOX3-positive cells in the cerebellar mantle zone do not develop into post-mitotic GABAergic Purkinje neurons. Purkinje neurons are instead derived from the PTF1a-expressing or Neph3-expressing and E-cadherin neuronal progenitors in the neuroepithelial cells of the cerebellum (Mizuhara et al. 2010). The role of SOX3 in the Purkinje cell lineage warrants further investigation.

In the P21 cerebellum, we found that the somata of these smaller SOX3-expressing cells are in juxtaposition with larger Purkinje cells; these SOX3-positive cells are in fact Bergmann glia (Yamada and Watanabe 2002). Bergmann glial cells are unipolar protoplasmic astrocytes that associate with migrating GCs as potential glia-guided neuronal migration in the developing cerebellum (Rakic 1971). Bergmann glial cells in the adult murine cerebellum have also been shown to express other molecular markers, including SOX1, SOX2, and SOX9; thus, Bergmann glial cells have been proposed to function as a third stem cell population in the adult brain of human and mouse (Sottile et al. 2006; Alcock et al. 2009), in addition to the NSCs in the subventricular zone of the cerebral cortex and in the subgranular zone of the dentate gyrus (Altman and Das 1965; Luskin 1993; Lois and Alvarez-Buylla 1994). In the P42 cerebellum (unpublished data), we did not detect SOX3-positive cells in the Purkinje cell layer, indicating that SOX3 does not play a role in maintaining the Bergmann glial cell population. Cerebellar glial cells are derived from the ventricular neuroepithelium. In the P7 cerebellum, we observed that virtually all SOX3-positive cells were co-expressed with either GFAP, SOX9, or SOX2, although not all SOX2-positive, SOX9-positive, and GFAP-positive glial cells were identified as SOX3-positive cells. These data indicate that the SOX3 protein is expressed exclusively in cerebellar glial-lineage, including a subset of glial cells.

We also found that cerebellar development was normal in the *Sox3* null mice. Given that *Sox1* and *Sox2* are also expressed in the VGE, it is possible that SoxB1 family members are functionally redundant in the cerebellum, as is thought to be the case for other regions in the CNS (Rizzoti et al. 2004). SoxB1 functional redundancy is also supported by the absence of Bergmann glia defects in the adult *Sox1* null cerebellum (Sottile et al. 2006). In humans, males born with hemizygous *Sox3* mutations do not exhibit gait or coordination defects, indicating that *Sox3* is not essential for cerebellar development (Laumonnier et al. 2002; Woods et al. 2005) likely due to functional redundancy (Alcock et al. 2009). Developing mice that lack multiple SoxB1 genes in the developing cerebellum may provide a useful strategy to investigate *Sox1/2/3* function in cerebellum. In addition, identification of *Sox3* target genes using ChIP-seq analysis, as has recently been demonstrated for neural progenitor cells (Bergsland et al. 2011), may be crucial to understanding *Sox3* function in the cerebellum at the molecular level.

## Conclusion

Our study aimed to determine the expression and function of the *Sox3* gene in the developing cerebellum. Immunohistochemistry revealed that SOX3 is expressed in the ventricular zone of the embryonic and postnatal cerebellum and that its expression is restricted to the cerebellar glial cell system during development and after birth. Further investigation of the cerebellar morphology of *Sox3* null mice suggested SOX3 expression is not required for cerebellar development, possibly due to the functional redundancy of other SoxB1 subgroup genes.

## Additional files

Additional file 1:
**Figure S1.** SOX3 is expressed in the embryonic neuroepithelial of the cerebellum. Sagittal sections of the cerebellum at E14.5 dpc. Arrows are showing the SOX3-positive cells that coexpressed with Ki67 (A), SOX2 (B), and GLAST (C). Additional file 2:
**Figure S2.** GFP signal is not detectable in postnatal cerebellum. Sagittal sections of the cerebellum at postnatal day-1 immunostained with GFP (A), Ki67 (B), DAPI (C), and GFP/DAPI (D). (A) GFP signals were not detectable in the postnatal cerebellum. Some GFP+ cells are expressed in cells from the inferior colliculus (IC) of the midbrain.

## References

[CR1] Alcock J, Sottile V (2009). Dynamic distribution and stem cell characteristics of Sox1-expressing cells in the cerebellar cortex. Cell Res.

[CR2] Alcock J, Lowe J, England T, Bath P, Sottile V (2009). Expression of Sox1, Sox2 and Sox9 is maintained in adult human cerebellar cortex. Neurosci Lett.

[CR3] Altman J, Das GD (1965). Autoradiographic and histological evidence of postnatal hippocampal neurogenesis in rats. J Comp Neurol.

[CR4] Bergsland M, Ramskold D, Zaouter C, Klum S, Sandberg R, Muhr J (2011). Sequentially acting Sox transcription factors in neural lineage development. Genes Dev.

[CR5] Brunelli S, Silva Casey E, Bell D, Harland R, Lovell-Badge R (2003). Expression of Sox3 throughout the developing central nervous system is dependent on the combined action of discrete, evolutionarily conserved regulatory elements. Genesis.

[CR6] Bylund M, Andersson E, Novitch BG, Muhr J (2003). Vertebrate neurogenesis is counteracted by So1–3 activity. Nat Neurosci.

[CR7] Collignon J, Sockanathan S, Hacker A, Cohen-Tannoudji M, Norris D, Rastan S (1996). A comparison of the properties of Sox-3 with Sry and two related genes, Sox-1 and Sox-2. Development.

[CR8] Denny P, Swift S, Brand N, Dabhade N, Barton P, Ashworth A (1992). A conserved family of genes related to the testis determining gene, SRY. Nucleic Acids Res.

[CR9] Joyner AL (1996). Engrailed, Wnt and Pax genes regulate midbrain—hindbrain development. Trends Genet.

[CR10] Laronda MM, Jameson JL (2011). Sox3 functions in a cell-autonomous manner to regulate spermatogonial differentiation in mice. Endocrinology.

[CR11] Laumonnier F, Ronce N, Hamel BC, Thomas P, Lespinasse J, Raynaud M, Paringaux C (2002). Transcription factor SOX3 is involved in X-linked mental retardation with growth hormone deficiency. Am J Hum Genet.

[CR12] Lee K, Tan J, Morris MB, Rizzoti K, Hughes J, Cheah PS (2012). Congenital hydrocephalus and abnormal subcommissural organ development in Sox3 transgenic mice. PLoS One.

[CR13] Lois C, Alvarez-Buylla A (1994). Long-distance neuronal migration in the adult mammalian brain. Science.

[CR14] Luskin MB (1993). Restricted proliferation and migration of postnatally generated neurons derived from the forebrain subventricular zone. Neuron.

[CR15] Mizuhara E, Minaki Y, Nakatani T, Kumai M, Inoue T, Muguruma K (2010). Purkinje cells originate from cerebellar ventricular zone progenitors positive for Neph3 and E-cadherin. Dev Biol.

[CR16] Rakic P (1971). Neuron-glia relationship during granule cell migration in developing cerebellar cortex. A Golgi and electronmicroscopic study in Macacus Rhesus. J Comp Neurol.

[CR17] Rex M, Orme A, Uwanogho D, Tointon K, Wigmore PM, Sharpe PT (1997). Dynamic expression of chicken Sox2 and Sox3 genes in ectoderm induced to form neural tissue. Dev Dyn.

[CR18] Rizzoti K, Brunelli S, Carmignac D, Thomas PQ, Robinson IC, Lovell-Badge R (2004). SOX3 is required during the formation of the hypothalamo-pituitary axis. Nat Genet.

[CR19] Rogers N, Cheah PS, Szarek E, Banerjee K, Schwartz J, Thomas PQ (2013). Expression of the murine transcription factor SOX3 during embryonic and adult neurogenesis. Gene Expr Patterns.

[CR20] Solomon NM, Nouri S, Warne GL, Lagerstrom-Fermer M, Forrest SM, Thomas PQ (2002). Increased gene dosage at Xq26-q27 is associated with X-linked hypopituitarism. Genomics.

[CR21] Sottile V, Li M, Scotting PJ (2006). Stem cell marker expression in the Bergmann glia population of the adult mouse brain. Brain Res.

[CR22] Stevanovic M, Lovell-Badge R, Collignon J, Goodfellow PN (1993). SOX3 is an X-linked gene related to SRY. Hum Mol Genet.

[CR23] Sutton E, Hughes J, White S, Sekido R, Tan J, Arboleda V (2011). Identification of SOX3 as an XX male sex reversal gene in mice and humans. J Clin Invest.

[CR24] Szarek E, Cheah PS, Schwartz J, Thomas P (2010). Molecular genetics of the developing neuroendocrine hypothalamus. Mol Cell Endocrinol.

[CR25] Uwanogho D, Rex M, Cartwright EJ, Pearl G, Healy C, Scotting PJ (1995). Embryonic expression of the chicken Sox2, Sox3 and Sox11 genes suggests an interactive role in neuronal development. Mech Dev.

[CR26] Wang VY, Zoghbi HY (2001). Genetic regulation of cerebellar development. Nat Rev Neurosci.

[CR27] Wang TW, Stromberg GP, Whitney JT, Brower NW, Klymkowsky MW, Parent JM (2006). Sox3 expression identifies neural progenitors in persistent neonatal and adult mouse forebrain germinative zones. J Comp Neurol.

[CR28] Wingate RJT, Hatten ME (1999). The role of the rhombic lip in avian cerebellum development. Development.

[CR29] Wood HB, Episkopou V (1999). Comparative expression of the mouse Sox1, Sox2 and Sox3 genes from pre-gastrulation to early somite stages. Mech Dev.

[CR30] Woods KS, Cundall M, Turton J, Rizotti K, Mehta A, Palmer R (2005). Over- and underdosage of SOX3 is associated with infundibular hypoplasia and hypopituitarism. Am J Hum Genet.

[CR31] Yamada K, Watanabe M (2002). Cytodifferentiation of Bergmann glia and its relationship with Purkinje cells. Anat Sci Int.

