# Supplementary material for: SOX3 expression in the glial system of the developing and adult mouse cerebellum
Source: Springerplus. 2015 Aug 7;4:400. doi: 10.1186/s40064-015-1194-1 (PMC4527974; doi:10.1186/s40064-015-1194-1)
Supplement: Additional file 1: — Figure S1. SOX3 is expressed in the embryonic neuroepithelial of the cerebellum. Sagittal sections of the cerebellum at E14.5 dpc. Arrows are showing the SOX3-positive cells that coexpressed with Ki67 (A), SOX2 (B), and GLAST (C). [file 40064_2015_1194_MOESM1_ESM.pdf]

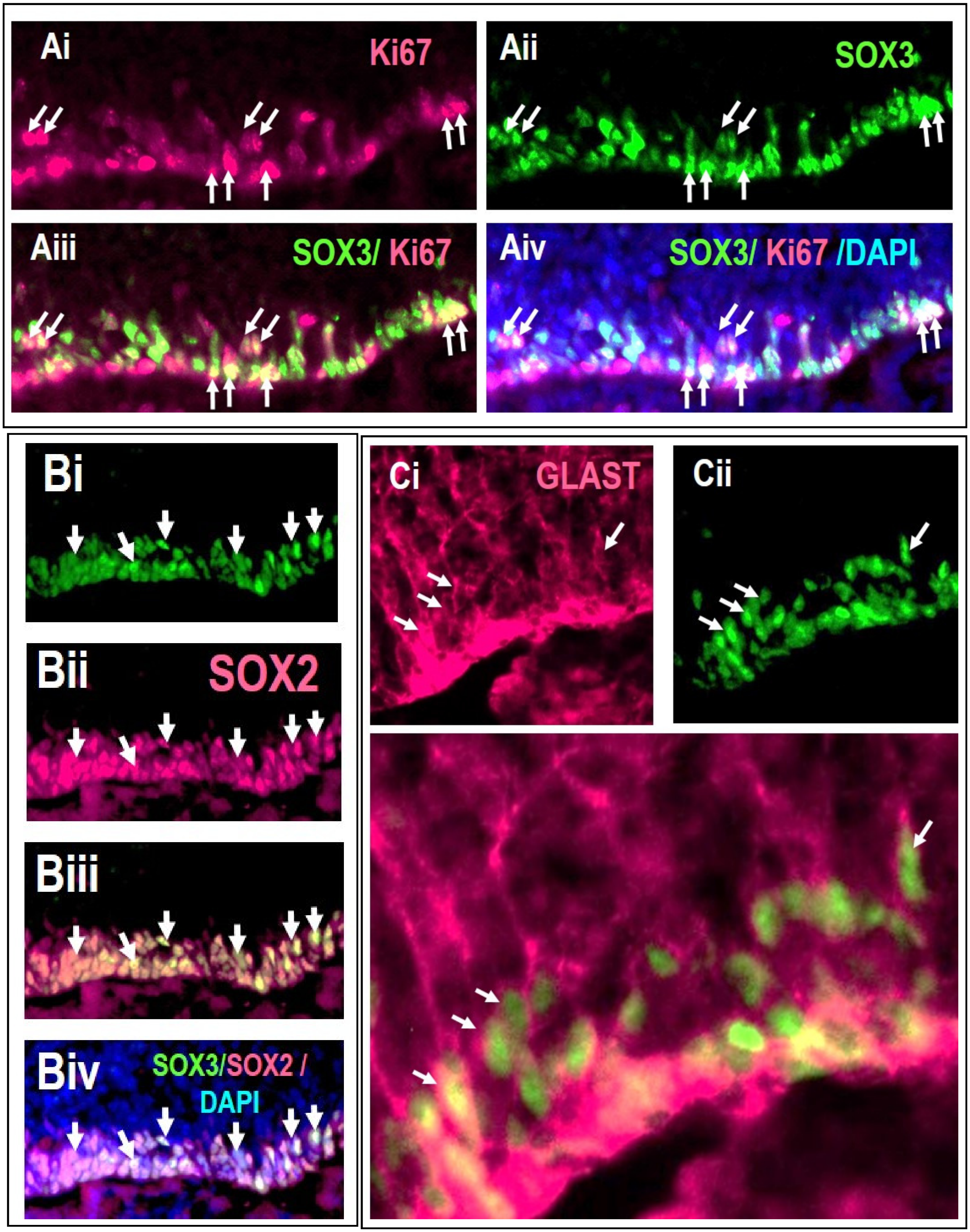

Supplementary Figure 1. SOX3 is expressed in the embryonic neuroepithelial of the cerebellum. Sagittal sections of the cerebellum at E14.5 dpc. Arrows are showing the SOX3-positive cells that coexpressed with Ki67 (A), SOX2(B), GLAST (C).
