# Supplementary material for: SOX3 expression in the glial system of the developing and adult mouse cerebellum
Source: Springerplus. 2015 Aug 7;4:400. doi: 10.1186/s40064-015-1194-1 (PMC4527974; doi:10.1186/s40064-015-1194-1)
Supplement: Additional file 2: — Figure S2. GFP signal is not detectable in postnatal cerebellum. Sagittal sections of the cerebellum at postnatal day-1 immunostained with GFP (A), Ki67 (B), DAPI (C), and GFP/DAPI (D). (A) GFP signals were not detectable in the postnatal cerebellum. Some GFP+ cells are expressed in cells from the inferior colliculus (IC) of the midbrain. [file 40064_2015_1194_MOESM2_ESM.pdf]

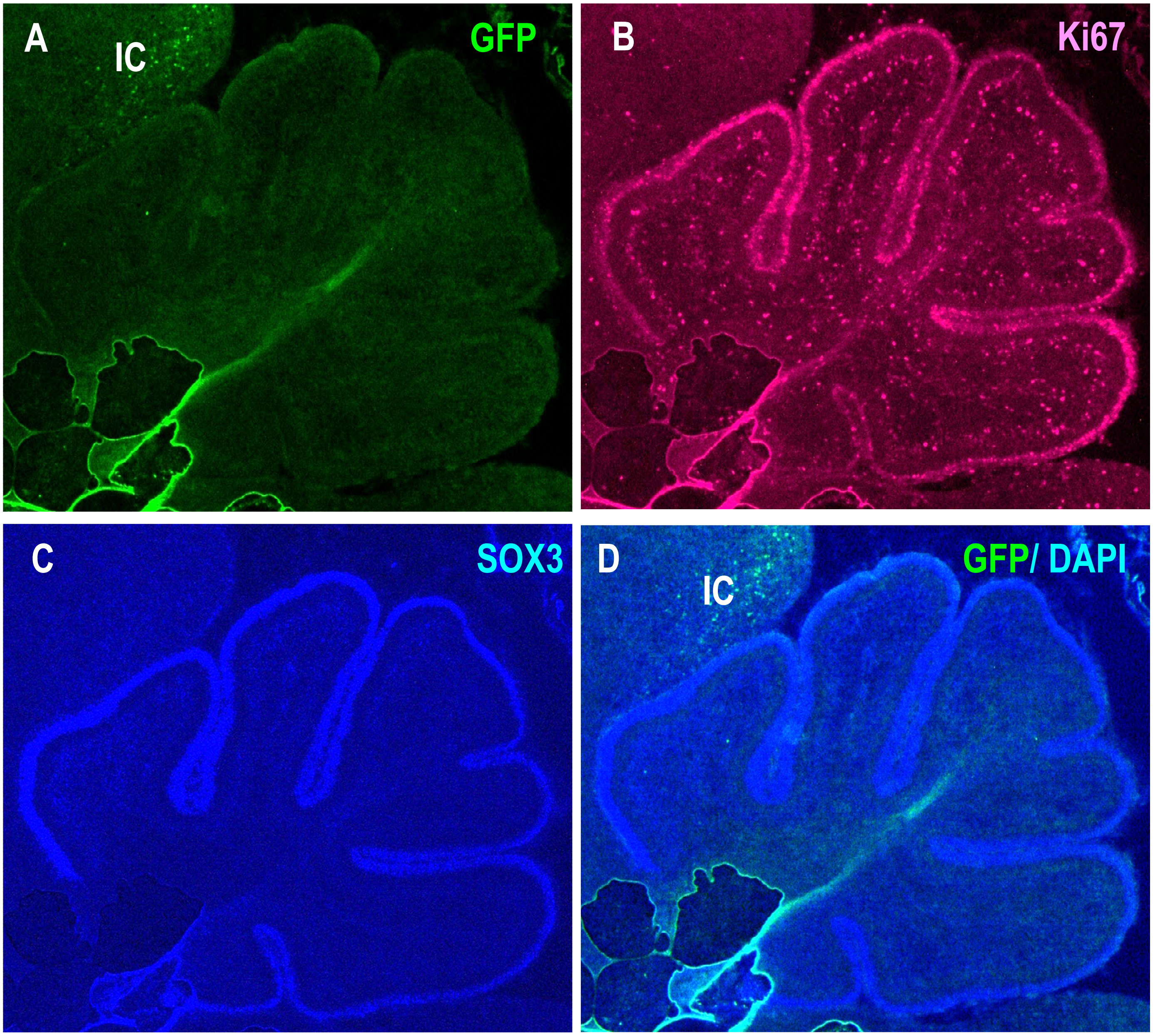

Supplementary Figure 1. GFP signal is not detectable in postnatal cerebellum. Sagittal sections of the cerebellum at postnatal day-1 immunostained with GFP (A), Ki67(B), DAPI(C) and GFP/DAPI (D). (A) GFP signals was not detectable in the postnatal cerebellum. Some GFP+ve cells are expressed with the brain cells from the inferior colliculus(IC) of the midbrain.
